# Multimodal Cleavable Reporters for Quantifying Carboxy and Amino Groups on Organic and Inorganic Nanoparticles

**DOI:** 10.1038/s41598-019-53773-3

**Published:** 2019-11-26

**Authors:** Nithiya Nirmalananthan-Budau, Bastian Rühle, Daniel Geißler, Marko Moser, Christopher Kläber, Andreas Schäfer, Ute Resch-Genger

**Affiliations:** 1 Federal Institute for Materials Research and Testing (BAM), Richard-Willstätter-Str. 11, D-12489 Berlin, Germany; 2 Institut für Chemie und Biochemie, Freie Universität Berlin, Takustrasse 3, 14195 Berlin, Germany

**Keywords:** Surface chemistry, Organic-inorganic nanostructures

## Abstract

Organic and inorganic nanoparticles (NPs) are increasingly used as drug carriers, fluorescent sensors, and multimodal labels in the life and material sciences. These applications require knowledge of the chemical nature, total number of surface groups, and the number of groups accessible for subsequent coupling of e.g., antifouling ligands, targeting bioligands, or sensor molecules. To establish the concept of catch-and-release assays, cleavable probes were rationally designed from a quantitatively cleavable disulfide moiety and the optically detectable reporter 2-thiopyridone (2-TP). For quantifying surface groups on nanomaterials, first, a set of monodisperse carboxy-and amino-functionalized, 100 nm-sized polymer and silica NPs with different surface group densities was synthesized. Subsequently, the accessible functional groups (FGs) were quantified *via* optical spectroscopy of the cleaved off reporter after its release in solution. Method validation was done with inductively coupled plasma optical emission spectroscopy (ICP-OES) utilizing the sulfur atom of the cleavable probe. This comparison underlined the reliability and versatility of our probes, which can be used for surface group quantification on all types of transparent, scattering, absorbing and/or fluorescent particles. The correlation between the total and accessible number of FGs quantified by conductometric titration, qNMR, and with our cleavable probes, together with the comparison to results of conjugation studies with differently sized biomolecules reveal the potential of catch-and-release reporters for surface analysis. Our findings also underline the importance of quantifying particularly the accessible amount of FGs for many applications of NPs in the life sciences.

## Introduction

Surface-functionalized organic, inorganic, and hybrid nanoparticles (NPs) are increasingly used in the life and material sciences^1–5^ with many foreseeable applications e.g., in medical and consumer products. Amongst the most frequently used NPs are polystyrene particles (PSP) and silica NPs. These NPs can be prepared in different sizes with different surface functionalities and commonly show a high colloidal stability, a remarkable stability in biological environments, and a good biocompatibility^6–9^. Moreover, they can be easily doped with functional molecules like fluorescent dyes and the large number of FGs at their surface makes them interesting carriers for stimuli-responsive sensor molecules and releasable drugs^10^. In addition, mesoporous silica nanoparticles (MSN) have the advantage of a particularly large surface-to-volume ratio, with uniform and tunable pore sizes^11–14^. These properties paved the road for applications of PSP and MSN as multichromophoric reporters for signal enhancement and multiplexing strategies in optical assays, targeted probes in bioimaging studies, and nanoscale sensors^9,15–21^.

For the majority of NP applications, NP performance depends mainly on particle size, size distribution, shape, surface chemistry, and charge. These features control colloidal stability, subsequent functionalization with ligands or biomolecules, and the interaction with the environment, and thereby, biocompatibility and toxicity^4,22,23^. Thus, important prerequisites for every commercial application of NPs are not only reproducible particle syntheses, but also suitable methods for characterizing these features. Particularly relevant for material processing and for monitoring batch-to-batch variation is the quantification of the total and accessible amounts of functional groups (FGs) at the NP surface^22,24^. This calls for robust, simple, and inexpensive methods that should preferably be validated. Additionally, these methods should be specific, sensitive, fast, and versatile, i.e., useable for characterizing a broad variety of particle systems independent of their chemical composition or optical properties such as scattering, absorption, and emission. Meanwhile, there exists a large toolbox of methods for FG analysis to choose from. Principally suited techniques range from X-ray photoelectron spectroscopy (XPS)^22^, (quantitative) nuclear magnetic resonance spectroscopy (NMR)^24,25^, thermogravimetric analysis (TGA) sometimes in conjunction with Fourier transform infrared spectroscopy (FTIR) or mass spectrometry (MS), as well as inductively coupled plasma optical emission spectroscopy (ICP-OES) and mass spectrometry (ICP-MS)^26^. These methods need, however, expensive instrumentation and are commonly operated by specifically trained personnel. Only very few methods can provide the total number of FGs. Examples are very versatile quantitative NMR (qNMR) techniques requiring special measurement conditions and specific signals distinguishable from those of the NP matrix, and inexpensive electrochemical titration methods like conductometry for (de)protonatable FGs like carboxyl and amino groups. The latter methods require, however, a relatively large amount of substance and unspecifically detect all (de)protonatable species present in solution which have comparable pK_a_ values, including initiators, stabilizers, and surfactants remaining from the particle preparation, or other (de)protonatable surface groups such as silanols^27–30^. Also, not all methods are suitable for all types of nanomaterials. For example, TGA with mass loss detection can only be used to determine the number of organic ligands, and thus, the number of FGs for one type of surface ligand, on inorganic particles^31^.

Elegant and frequently used methods to quantify the number of accessible surface groups are optical assays with spectrophotometrical or fluorometrical readout. These assays typically require a labeling step, i.e., the covalent binding of an optically detectable reporter bearing a reactive group. Quantification can be done with commonly used and widely available laboratory instrumentation such as spectrometers or microplate readers using a calibrant with known optical features closely matching those of the species detected in the respective assay. Optical measurements, however, can be affected by the absorption and fluorescence properties of the (nano)material to be analyzed, dye-dye interactions leading to fluorescence quenching, and the inherent environment sensitivity of the spectroscopic features of the optical reporters used for quantification, namely the reporter´s molar absorption coefficient and its fluorescence quantum yield. The consideration of these effects can impose challenges on assay calibration. To overcome some of these challenges, we recently presented the versatile concept of catch-and-release reporters, utilizing as first examples *N*-(aminomethyl)-3-(pyridin-2-yldisulfanyl)propanamide (*N*-APPA) and *N-*Succinimidyl-3-(2-pyridyldithio) propionate (SPDP, NHS-PDP) for quantifying carboxy and amino groups on nano- and microparticles^32^. These cleavable probes consist of reactive groups for the coupling to surface functionalities *via* established conjugation chemistries, a quantitatively cleavable unit like in our case a reductively cleavable disulfide moiety, and an optically detectable reporter. Here, 2-thiopyridone (2-TP) was chosen containing a sulfur heteroatom that can also be quantified by complementary analytical techniques such as ICP-OES or ICP-MS. Cleavage of the disulfide unit releases 2-TP which can then be detected in the supernatant photometrically or with ICP-OES. Thereby, the distortion of optical signals by particle scattering is avoided and simple method validation by method comparisons and mass balances can be obtained^32,33^.

**Figure 1:**
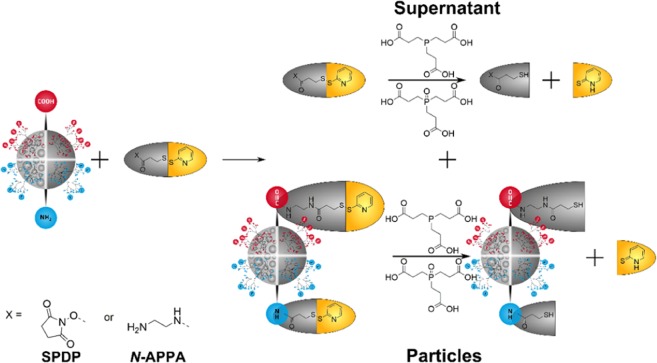
Mechanism for quantifying the accessible amount of carboxy and amino groups on PSP and MSN with the cleavable reporters *N*-APPA and SPDP, respectively.

To demonstrate the potential of our reductively cleavable probes for the quantification of some of the most frequently used particles and surface FGs in life science applications, we prepared a series of PSP and MSN, surface modified with varying densities of carboxyl and amino groups. These FGs were then quantified with conductometric titration, qNMR, and optical assays using the custom-designed cleavable probes *N*-APPA and SPDP shown in Fig. 1. To underline the versatility of our catch-and release reporter concept, we also performed similar studies with dye-loaded PSP. Additionally, the results were correlated with data from PSP conjugation to differently sized biomolecules. Here, the proteins bovine serum albumin (BSA) and streptavidin (SAv), as well as wheat germ agglutinin (WGA) and concanavalin A (ConA), two lectins often used for the design of bioconjugates for bacteria detection^34,35^, were chosen exemplarily due to their different protein size ranging from 25 to 67 kDa.

## Experimental

### Materials

Styrene, potassium persulfate (PPS), sodium dodecyl sulfate (SDS), acrylic acid (AA), tetraethyl orthosilicate (TEOS), (3-aminopropyl)triethoxysilane (APTES), cetyltrimethylammonium bromide (CTAB), ammonium nitrate (NH_4_NO_3_), 1-ethyl-3-(3-dimethylaminopropyl)carbodiimide (EDC), *N*-hydroxysuccinimide (NHS), tetrahydrofuran (THF), dimethyl sulfoxide (DMSO), methanol (MeOH), ethanol (EtOH), sodium dihydrogen phosphate, *N*-Boc-ethylendiamine (EDA), sodium hydroxide (NaOH), and hydrochloric acid (HCl) were obtained from Sigma Aldrich Co. (Germany, www.sigmaaldrich.com/germany), *N-*Succinimidyl-3-(2-pyridyldithio) propionate (SPDP), tris(2-carboxyethyl) phosphine hydrochloride (TCEP) were purchased from Thermo Fisher Scientific Germany BV & Co KG (Germany, www.thermofisher.com/de/de/home), 2-cyanoethyltriethoxysilane (CETES) was obtained from abcr (Germany, www.abcr.de). All solvents used in optical assays were of spectroscopic grade. All solutions and buffers were prepared with Milli-Q water (Millipore).

### Synthesis of functionalized PSP and MSN

The detailed synthetic procedures for the formation of the carboxylated PSP and MSN as well as the aminated PSP and MSN is described in the Supporting Information (SI) together with the basic characterization of the produced particles regarding particle size/size distribution, surface charge, nitrogen sorption data, and thermogravimetric analysis (TGA).

### Activation of carboxy groups on PSP and MSN

Activation of carboxy groups was carried out as previously described in Moser & Nirmalananthan *et al*.^32^. Briefly, 120 µL of EDC (150 mM) and 60 µL of NHS (300 mM) dissolved in phosphate buffer (0.01 M; pH 5.5) were added to 100 µL of carboxy PSP or MSN (5 wt-%) in phosphate buffer. The mixture was shaken at 700 rpm for 1 h at room temperature (RT), followed by one washing step using 300 µL of phosphate buffer (0.01 M, pH 8 for PSP and pH 7.4 for MSN).

### Quantification of carboxy groups on PSP and MSN with *N*-APPA

The quantification of carboxy groups was performed according to Moser & Nirmalananthan *et al*.^32^. Briefly, 580 µL of the activated carboxy particle (0.86 wt-%) were mixed with a solution of 1.65 µmol (for PSP) or 2.5 µmol (for MSN) *N*-APPA which was dissolved in 20 µL of methanol. The mixture was shaken at 700 rpm for 16 h at RT, followed by centrifugation at 16,000 g for 40 min for PSP and 10,000 g for 5 min for MSN. The supernatant was collected, and the *N*-APPA-coupled particle suspension was washed three times with 400 µL of phosphate buffer (0.01 M, pH 8 for PSP and pH 7.4 for MSN). 50 µL of TCEP solution (40 mM) in phosphate buffer was added to the combined supernatant solutions containing the unreacted *N*-APPA (pH of the resulting PSP supernatant solution 6.8) and shaken at 600 rpm for 45 min at RT. The cleaved-off 2-TP was quantified spectrophotometrically at λ_abs_ = 343 nm (ε = (8000 ± 100) M^−1^cm^−1^).

Additionally, 50 µL of TCEP was added to the washed particles (PSP, MSN) resuspended in 350 µL of phosphate buffer (pH of the resulting PSP suspension 3.8). The particle suspension was washed twice with phosphate buffer after shaking it at 700 rpm for 45 min at RT. The 2-TP amount in the second supernatant was also determined spectrophotometrically. The number of thiol groups in all fractions were obtained with ICP-OES.

### Quantification of amino groups on PSP and MSN with SPDP

Following a previously reported protocol^32^ aminated PSP or MSN (0.86 wt-%) suspended in 580 µL of phosphate buffer (0.01 M, pH 8 for PSP and pH 7.4 for MSN) were added to a solution of 2 µmol of SPDP which was dissolved in 20 µL of DMSO. After shaking the sample at 700 rpm for 45 min at RT, it was centrifugated at 16,000 g for 40 min for PSP and 10,000 g for 5 min for MSN. The supernatant was collected separately, and the particle suspensions were washed three times with 400 µL of phosphate buffer (0.01 M, pH 8 for PSP and pH 7.4 for MSN). The combined supernatant solutions containing unreacted SPDP were mixed with 50 µL of TCEP solution (40 mM) in phosphate buffer and shaken at 700 rpm for 45 min at RT. The cleaved-off 2-TP was quantified spectrophotometrically at λ_abs_ = 343 nm (ε = (8000 ± 100) M^−1^cm^−1^).

Further, 50 µL of TCEP solution was added to the washed particles (PSP and MSN) resuspended in 350 µL of phosphate buffer. After shaking at 700 rpm for 45 min at RT, the particle suspension was washed twice with phosphate buffer. The 2-TP in the combined supernatants was quantified spectrophotometrically.

### Dye staining of PSP

Carboxy-functionalized PSP with a high FG density (PSP-COOH-Hi) were stained exemplary with the hydrophobic dyes phenyl-pyrrolidinylvinylquinoxaline (PVQ), coumarin 153, and Nile Red according to a previously reported procedure from Behnke *et al*.^17^ and Nirmalananthan *et al*.^36^. Briefly, 400 µL of a 6 mM dye solution in THF were mixed with 2400 µL of 12 mg of PSP suspension (0.5 wt-%). After shaking for 30 min at RT, the dye-encapsulated PSP were washed three times with Milli-Q water and subsequently resuspended under sonication. Afterwards, the PSP were washed with an ethanol−water mixture (50/50 v/v) and re-suspended in phosphate buffer (0.01 M, pH 5.5).

The quantification of the accessible amount of FGs on the dye-loaded PSP was done following the procedure for undoped carboxy PSP and MSN described above (see sections on *Activation of carboxy groups on PSP and MSN* and *Quantification of carboxy groups on PSP and MSN with N-APPA*).

### Biomolecule coupling

After the activation of PSP-COOH-Hi (see section on *Activation of carboxy groups on PSP and MSN*), 275 µL of bovine serum albumin (BSA), streptavidin (SAv), wheat germ agglutinin (WGA), or concanavalin A (ConA) in phosphate buffer (pH 7.4) containing 50 nmol of protein were added to 125 µL of a suspension of 5 mg of carboxy PSP (4 w%) in phosphate buffer. The reaction mixture was shaken at RT overnight, followed by centrifugation. The supernatant was collected, the carboxy PSP were washed four times with 300 µL of phosphate buffer, all supernatants were combined, and then used for quantifying the amount of unreacted biomolecules.

### BCA assay

The amount of biomolecules covalently bound to the carboxy PSP and unreacted biomolecules in the supernatant were determined with the BCA assay, using. 25 μL of the 2.5-fold diluted supernatant and 25 µL of a 2.5-fold diluted 5 wt-% suspension of biomolecule-labeled PSP. 200 μL of BCA reagent were added to these biomolecule-containing solutions at RT and shaken for 120 min. To avoid signal distortions from particle scattering, the samples were centrifuged and 225 µL of the supernatants were added to a microtiter plate. The amount of biomolecules in all wells was quantified photometrically at λ_abs_. = 562 nm by comparison with the results from the calibration curve, obtained with nine biomolecule standards (25 µL each) in a concentration range of 0 to 2000 µg∙mL^−1^. The average biomolecule labeling density per mg of particles was calculated from the results of the BCA assay and the amount of particles used in the assay.

### Instrumentation

#### Dynamic light scattering (DLS) and zeta potential measurements

The measurements were done with a Zetasizer Nano ZS instrument from Malvern Instruments Ltd. and the data were analyzed with the Zetasizer Software v7.02. All samples were measured at T = 25 °C using PS-semi-micro cuvettes from ratiolab.

#### Transmission scanning electron microscopy (T-SEM)

T-SEM images were acquired on a Hitachi SU8030 EM. Images were taken with an acceleration voltage of 30 kV, in transmission electron (TE) mode. T-SEM images were used for the size analysis of the particles using the program FIJI.

#### Absorption measurements

Absorbance spectra were recorded using a Specord 210 plus spectrophotometer from Analytik Jena (Germany) in (10 × 10) mm quartz cuvettes from Hellma GmbH (Germany) at RT. For the BCA assay, absorption spectra were recorded with a Microplate Reader Infinity M200 Pro from Tecan Inc. (Austria) at RT in microtiter plates from Fischer Scientific GmbH (Germany).

#### ICP-OES measurements

Quantitative determination of the sulfur content in the different fractions (applied, unreacted, cleaved off from the particles) was measured with a 5110 ICP-OES from Agilent Technologies. The sulfur signal at 180.669 nm was used for the quantification.

The relative standard deviations of all optical assays were obtained from six independent measurements. For ICP-OES measurements, the relative standard deviations were determined from three independent measurements.

#### Conductometric titration

Conductivity measurements giving the amount of (de)protonatable FGs were measured with a Modul 856 conductometer (Metrohm) at RT following a previously described presedure^32^. For complete protonation/deprotonation of the FGs on the particle surface, the conductivity of the suspensions was adjusted to 100 μS/cm with HCl or NaOH before to FG titration. The samples containing 10 mg of PSP-COOH or 5 mg of PSP-NH_2_ in 80 mL of Milli-Q water were titrated with 10 mM NaOH or 10 mM HCl in 20 µL steps under argon atmosphere.

#### qNMR

Solution ^1^H NMR spectra were collected at RT on a JEOL JNM-ECZ600R/M1 spectrometer operated at 600 MHz or on a Bruker Avance III HD 500 operated at 500 MHz. The MSN samples were prepared by drying a known amount (approximately 20 mg) at 80 °C for 24 h, adding a known amount (approximately 1–2 mg) of 1,3,5 trioxane as an internal standard, and dissolving the mixture in 1 mL of NaOD in D_2_O (1 M, pH 14) under sonication for 30 min at RT^37^. Prior to collecting qNMR spectra, the T1 times of the components were measured using a series of inversion-recovery experiments. For the qNMR spectra, single 90° pulses with an interpulse delay of at least seven times the longest T1 proton relaxation time (9.2 s for the methylene protons of trioxane in 1 M NaOD) and 128 scans (for samples with high FG density) or 512 scans (for samples with low FG density) were used. Prior to fast Fourier transformation (FFT), the free induction decays (FIDs) were multiplied with an exponential window function (line broadening 0.1 Hz). Transformed spectra were referenced to the residual solvent proton signal centered at 4.79 ppm (broad singlet)^38^, the phase was adjusted manually, and baseline-corrected integral values for the methylene protons of the silane groups and the internal standard were calculated. The purity of the internal standard was determined to be 96% by measuring it against 1,3,5-trimethoxybenzene of known purity (99%) in CDCl_3_ (known amount of approximately 5 mg each in 1 mL of CDCl_3_, 4 scans, 90° single pulse, interpulse delay of 20 s, other data processing parameters as described above).

#### Nitrogen sorption measurements

Nitrogen adsorption and desorption isotherms were recorded on an ASAP 2020 from micromeritics at liquid nitrogen temperature (77 K). Approximately 20 mg of sample were degassed for 16 h at 120 °C under vacuum. BET surface areas were calculated from the linear part of the BET plots (typically in a partial pressure range from ~0.1–0.3), giving correlation coefficients of at least 0.999). BJH and NLDFT pore size distributions were calculated from the desorption branch, using an equilibrium model for N_2_ on silica for NLDFT, as implemented in the Quantachrome ASiQwin software v 3.01.

#### Thermogravimetric analysis (TGA)

The measurements were carried out on a Perkin-Elmer TGA 7 in synthetic air using approximately 5 mg of sample. The samples were heated from RT to 120 °C with a heating rate of 5 K/min, held at 120 °C for 60 min, and then heated from 120 °C to 700 °C with a heating rate of 5 K/min. The weight loss is normalized to the sample weight obtained after 60 min at 120 °C.

## Results and Discussion

### Synthesis and characterization of carboxyl and amino PSP and MSN

Carboxy PSP with different FG densities were synthesized by an emulsion polymerization reaction following a slightly modified procedure from Holzapfel *et al*.^39^ by adding different amounts of co-monomer AA. The size of the particles was determined with DLS to be between 100 nm and 150 nm (intensity-weighted harmonic mean diameter, so-called Z-average). The monodisperse, spherical, ~100 nm-sized particles have a zeta potential of −25 mV and −48 mV in Milli-Q water (pH 7) for the samples with low functionalization density (PSP-COOH-Lo) and high functionalization density (PSP-COOH-Hi), respectively. The aminated PSP were synthesized by derivatization of PSP-COOH-Hi with three different amounts of EDA. This derivatization, which is also used in the synthesis of many commercialized aminated particles, results in an increase in PSP diameter by approximately 15 nm and an increase of the zeta potential from −48 mV for PSP-COOH-Hi to −25 mV for PSP-NH2-Hi.

Carboxylated and aminated MSN were prepared according to the literature^40^, by a slightly modified surfactant-templated (CTAB) sol-gel method using the co-condensation of TEOS and different amounts of CETES or APTES (10 mol% or 0.1 mol% with respect to TEOS). The resulting particles have intensity-weighted Z-average hydrodynamic diameters of 200 nm and 320 nm, respectively. The carboxy MSN have negative zeta potential values of −25 mV (MSN-COOH-Lo) and −15 mV (MSN-COOH-Hi) in Milli-Q water (pH 7). The respective aminated MSN have positive zeta potentials of +34 mV (MSN-NH_2_-Lo) and +41 mV (MSN-NH_2_-Hi). All synthesis procedures and characterization results of all particles used in this study are given in detail in the Supporting information (SI) in Figs. S1–S7 and in Tables S1–S3.

### Quantification of total carboxy and amino groups on PSP by conductometric titration

This method was previously validated by us for the quantification of carboxy groups on differently sized, surface modified polymethylmethacrylate (PMMA) micro- and nanoparticles grafted with poly(acrylic acid) by comparison with qNMR^24^ and for aminated particles by a Fluram assay^32^. The results obtained for the carboxylated and aminated PSP are summarized in Table 1. This table reveals that the increasing volume of the co-monomer AA which was added during the *in-situ* co-polymerization leads to an increase of the carboxy FG density on the PSP surface.

**Table 1:** Quantification of total and accessible amount of surface FGs on PSP and MSN.

	Surface density	Total FGs [µmol/g]	Accessible FGs [µmol/g]	(Accessible/Total) *100 [%]
PSP-COOH	Low	185 ± 36	27 ± 5	15
	Medium	393 ± 5	61 ± 6	16
	High	642 ± 14	85 ± 8	13
PSP-NH_2_	Low	621 ± 44	38 ± 4	6
	Medium	671 ± 60	52 ± 2	8
	High	686 ± 45	65 ± 1	10
MSN-COOH	Low	65	19 ± 4	29
	High	860	50 ± 5	6
MSN-NH_2_	Low	44	21 ± 7	46
	High	1532	219 ± 13	13

The aminated PSP which were made by derivatization of PSP-COOH-Hi with different amounts of EDA all show similarly high amounts of FGs, with the measured values being comparable to the total amount of carboxy FGs on unmodified PSP-COOH-Hi. Since during the derivatization process only a few accessible carboxy groups are aminated, these particles bear a mixture of carboxy and amine FGs on their surface, and the conductometric titration is not sensitive enough to distinguish between the (de)protonation of carboxy and amino groups. Hence, only a sum of all (de)protonatable FGs can be obtained (see Fig. S8). The corresponding value does not reflect the increase in amino FG density or the total amount of amino groups but can solely be used for the comparison of different PSP batches. Similarly, the quantification of the total amount of carboxy and amino FGs on MSN by conductometric titration is impeded by the presence of (de)protonatable silanol groups on the silica surface. The pK_a_ value of the silanol groups is about 4.5–5.5^41^ and the pK_a_ values for the carboxy and amino FGs are estimated to be at around 4.8 and 9–11, respectively. Additionally, the silica framework of the MSN can be hydrolyzed under basic conditions under the consumption of hydroxide ions. Both effects lead to an overestimation of the actual FG density (see Fig. S9). Therefore, the total amount of carboxy and amino FGs on MSN was quantified by qNMR rather than conductometry.

### Quantification of total carboxy and amino groups on MSN with qNMR

For the determination of the total number of FGs by qNMR, the MSN were dissolved in 1 M NaOD in D_2_O together with a known amount of an internal standard, here 1,3,5 trioxane, and the amount of carboxy and amino groups was determined from the ratio of the integrals of the methylene protons of trioxane and the methylene protons of the alkyl silane bearing the respective FGs (see SI, Figs. S10–S13). This procedure assumes that each alkyl chain corresponds to one FG^37,42^.

The total amount of amino FGs determined by qNMR agrees well with the expected amount of FGs calculated from the molar ratios of TEOS to APTES that were used for MSN synthesis. Assuming a stoichiometric reaction of TEOS and APTES and complete hydrolysis and condensation of the precursors, we expect FG densities of 1413 µmol/g and 17 µmol/g for samples MSN-NH_2_-Hi and MSN-NH_2_-Lo, respectively. The values measured by qNMR were 1532 µmol/g and 44 µmol/g for MSN-NH_2_-Hi and MSN-NH_2_-Lo (see Table 1). For carboxyl functionalized MSN, the difference between the expected and found amounts of FGs is somewhat larger. Under the same assumptions as above, as well as assuming a quantitative hydrolysis of nitrile groups to carboxy groups (which appears to be a good presumption given the very weak cyanoethylsilane signals visible in the ^1^H NMR spectra in Figs. S12 and S13), the expected FG densities are 1382 µmol/g and 17 µmol/g for samples MSN-COOH-Hi and MSN-COOH-Lo, while the amounts obtained from qNMR are 860 µmol/g and 65 µmol/g, respectively. A reason for the higher discrepancies could be – besides the simplifying nature of the assumptions – that some functional groups are hydrolyzed off and dissociate from the silica framework under the rather harsh nitrile hydrolysis conditions (5 h reflux in 9 M HCl). Interestingly, the carboxylated MSN samples also show lower BET surface areas, lower pore volumes, and smaller pore diameters than the aminated MSN samples (see Table S3 and Figure S6). This observation could be attributed to different effects of APTES and CETES during the synthesis by co-condensation. While the differences are noteworthy, a more thorough investigation is beyond the scope of the current work. TGA data confirms a higher weight loss of the samples with high FG density as compared to the samples with low FG density. Due to an unknown (and also variable) amount of volatiles such as H_2_O and EtOH present in the highly porous samples, an exact quantification of the FG density from TGA proved challenging. However, the differences in weight loss between the samples with high and low FG density were found to be similar for aminated and carboxylated MSNs, i.e., 3.5 and 3.7 wt-%, respectively.

### Quantification of accessible carboxy and amino groups on PSP

#### Carboxy PSP

**Figure 2:**
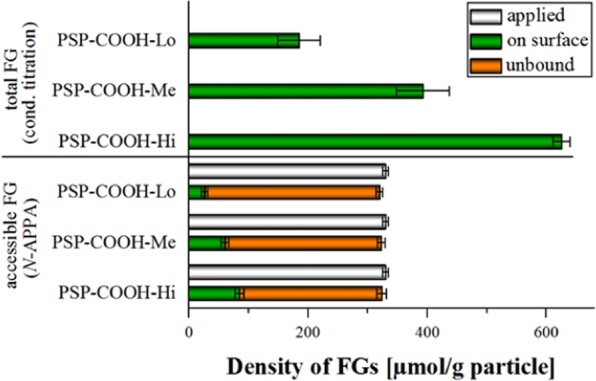
Quantification of FGs on PSP with different carboxy group densities. The total FG amount was measured by conductometric titration and the accessible FG amount was detected with the cleavable reporter *N*-APPA. Both methods can clearly distinguish between the different FG densities.

The amount of FGs on carboxy PSP with varying FG densities was quantified spectrophotometrically after reductive cleavage of the multimodal cleavable reporter *N*-APPA with TCEP and validated by quantifying the amount of sulfur by ICP-OES. First, *N-*APPA was coupled to the NHS activated carboxy PSP *via* its primary amino group. The 2-TP reporter, formed upon reductive cleavage of the disulfide bond with TCEP was used to quantify both the initially PSP-bound and the unreacted *N-*APPA molecules in the supernatant. The amount of carboxy groups accessible for *N-*APPA equals the amount of 2-TP cleaved off from the PSP surface. The results of the optical assays are summarized in Fig. 2 and in Table 1.

This method reveals that 15% (27 ± 5 µmol/g), 16% (61 ± 6 µmol/g) and 13% (85 ± 8 µmol/g) of the total amount of carboxy groups of PSP-COOH-Lo, PSP-COOH-Me, and PSP-COOH-Hi can be labeled with *N*-APPA, and thereby quantified. As expected, while the absolute amount of carboxy FGs on PSP-COOH-Hi is highest, the relative percentage of the accessible FGs is lower than that of PSP samples with lower FG densities. This underlines that a high FG density – albeit beneficial for the colloidal stability of the particles – must not necessarily be equally advantageous for the number of derivatizable FGs, where also steric effects and the size and charge of the molecules to be covalently attached come into play.

**Figure 3:**
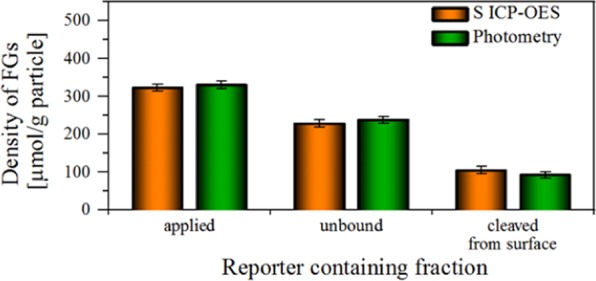
Validation of optical surface group analysis with *N*-APPA using ICP-OES exemplarily for PSP-COOH-Hi. A good correlation of both methods is observed.

The results of our spectrophotometric detection of 2-TP are in good agreement with those obtained with ICP-OES (see Fig. 3 and SI, Fig. S14).

#### Amine PSP

Quantification of amino groups was done with the cleavable reporter SPDP closely related to *N*-APPA, that bears an active NHS-ester group for the coupling reaction with the surface amino groups. The optical measurements of the reductively cleaved 2-TP reporter yielded 6%, 8%, and 10% of accessible amino groups (see Table 1 and Fig. S15) for PSP-NH_2_-Lo, PSP-NH_2_-Me, and PSP-NH_2_-Hi, respectively. Hence, an increase in the amount of EDA reacted with PSP-COOH-Hi clearly leads to the introduction of an increasing number of amino groups accessible for our cleavable probe. This number is, however, most likely lower than the total number of amino groups which was not determined here due to the previously discussed problems of conductometric titration to distinguish between carboxy and amino FGs. In summary, the results obtained with carboxylated and aminated PSP underline the suitability of our custom-made multimodal cleavable reporters *N*-APPA and SPDP for reliably quantifying the amount of accessible FGs on polymer nanoparticles.

### Quantification of accessible carboxy and amino groups on MSN

Next, our cleavable reporters *N*-APPA and SPDP were used for quantifying the amount of accessible carboxy and amino groups on another broadly used nanomaterial, MSN. For MSN, the assays with *N*-APPA and SPDP were performed in a less basic phosphate buffer at pH 7.4 to avoid particle etching or dissolution during the assay. As for PSP-COOH, the validation of the optical results for MSN-COOH was done by quantifying the amount of cleaved off 2-TP and the amount of unbound 2-TP in the supernatant with sulfur ICP-OES.

#### Carboxy MSN

**Figure 4:**
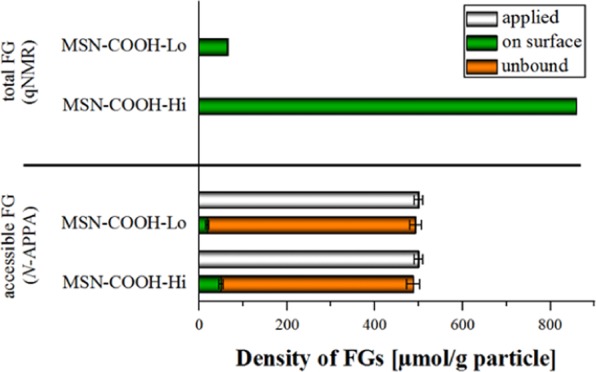
FG quantification on MSN-COOH-Lo and MSN-COOH-Hi using qNMR for the determination of the total amount and cleavable reporter *N*-APPA for the quantification of the accessible amount of FGs. FG density differences are clearly detectable with both methods.

The results obtained for the two carboxy MSN are summarized in Fig. 4 and in Table 1. As expected, the overall amount of accessible FGs in sample MSN-COOH-Hi is higher than in MSN-COOH-Lo. The percentage of accessible FGs on MSN-COOH-Hi and MSN-COOH-Lo was measured to be 6% and 29% of the total amount of FGs on MSN-COOH-Hi and MSN-COOH-Lo, respectively. The validation of optical results by ICP-OES (see SI, Fig. S16) also correlates well with the results from our optical assays for MSN.

The steric effects limit the number of covalently attachable probe molecules at high FG densities. It is also obvious from these results that the total amount of FGs as determined by qNMR is much higher than the amount of accessible FGs derived from the catch-and-release assay. Since a co-condensation approach was used to synthesize the functionalized MSN, we expect the FGs to be evenly distributed throughout over the particle surface. It is, however, possible that some FGs point into the silica pore walls rather than into the void of the mesopores or away from the outer surface, and hence, are not accessible for functionalization. Furthermore, while the pore size of the MSN is large enough to accommodate the cleavable reporter molecules, we assume that the reporters encounter first the FGs on the NP surface or the orifices of the mesopores and then react preferentially with those. This can lead to a diffusional barrier that prevents or at least significantly slows down the diffusion of further probe molecules deeper into the mesopores where they could react with further FGs.

#### Amino MSN

Reaction of MSN-NH_2_-Hi and MSN-NH_2_-Lo with SPDP yielded 13% and 46% of accessible amino groups (see Table 1 and Fig. S17). Here, again, the same trend is observed as for the other particles studied before. The well-matching mass balances, i.e., the fact that almost 100% of the initially used amount of the probe can be recovered either in its unbound form after the attachment step in the supernatant or after reductive cleavage from the particles, demonstrates that only a negligible amount of the reporter dye remains adsorbed to the large surface area of the porous structure, and hence, that our assays are also well-suited for porous host materials.

### Quantification of accessible carboxy groups on fluorescent PSP with *N*-APPA

**Figure 5:**
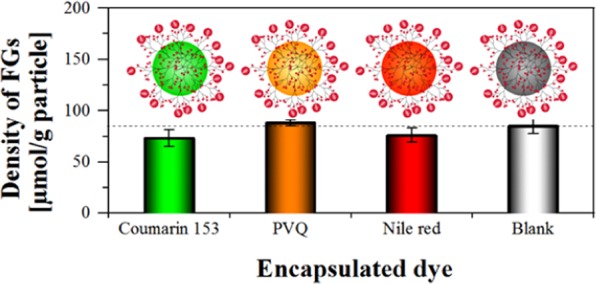
Accessible FG quantification of dye encoded carboxy PSP with cleavable reporter *N*-APPA.

NPs used in the life sciences are often colored or fluorescent. Since conventional FG quantification methods that rely on dye labeling of NP surface FGs are hampered by light scattering or other optical interferences from the particles as well as possible fluorescence quenching interactions between neighboring reporters at the NP surface, a complete dissolution of the PSP matrix can be necessary prior to quantifying the FG-bound reporter dyes^32^. However, for stained particles this simultaneously leads to the release of the dyes encapsulated in the NP. This can result in spectral interferences in absorption and/or emission with the reporter dye used for FG quantification, and thus, limiting the versatility of this approach (see SI, Fig. S18). In contrast, our versatile concept of catch-and-release assays is not affected by such interferences, as the quantification of both the unbound and the bound reporter are done in the supernatant after cleavage and particle separation. To demonstrate this absence of influence, we incorporated different hydrophobic fluorophores into PSP-COOH-Hi by a simple swelling/deswelling staining procedure that is also used for the fabrication of commercialized colored and fluorescent particles. This included frequently used dyes such as coumarin 153 and Nile Red^18,43^ as well as the PVQ derivative phenyl-pyrrolidinylvinylquinoxaline, a new dye revealing aggregation-induced emission (AIE)^36^. As shown in Fig. 5, the accessible amount of carboxy groups could be still determined with *N*-APPA for the three dye-loaded PSP-COOH-Hi, and the obtained FG densities agree reasonably well with the FG density determined for the corresponding undoped (blank) PSP-COOH-Hi.

### Correlation with bioconjugation studies

**Figure 6:**
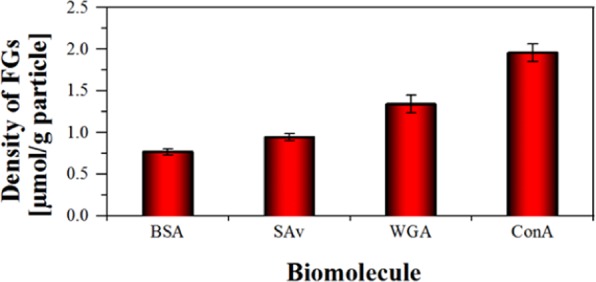
Amount of carboxy FGs accessible for bioconjugation of the differently sized proteins BSA, SAv, WGA, and ConA.

Four differently sized biomolecules were coupled to the surface of 100 nm-sized PSP-COOH-Hi *via* the amine groups of their lysine residues or the N-terminus after activation of the PSP with EDC/NHS. The exemplarily chosen proteins were BSA (67 kDa), often used as a model protein, SAv (53 kDa), employed for the directed conjugation of biotinylated molecules to NPs, and the smaller lectins WGA (36 kDa) and ConA (25 kDa) that are used, for example, for the staining of bacteria in conjunction with fluorescent labels^34,35^. The BCA assay was used to quantify the amount of PSP-bound proteins and the amount of unreacted biomolecules in the supernatant after particle removal. The amount of FGs accessible for the covalent binding of such large and sterically demanding biomolecules is typically below 1% of the total FG amount on PSP with a high FG density. The results of our bioconjugation studies are shown in Fig. 6. The mass balances, obtained from measurements of the unreacted biomolecules in the supernatant and the bound biomolecules on the PSP, and the coupling efficiencies of the biomolecules are detailed in the SI, Fig. S19.

As shown in Fig. 6, the amount of NP-bound biomolecules, and hence, the amount of carboxy surface FGs accessible for bioconjugation clearly depends on biomolecule size. For example, while 1.9 µmol/g of ConA could be bound to the particle surface, only 0.7 µmol/g of the largest biomolecule BSA are coupled to the surface, i.e., 2.9 times less. Moreover, the total amount of carboxy functions is 338 times higher and the accessible amount as determined with *N*-APPA is 42.5 times higher than the amount of FGs available for the binding of the smallest protein of this series, ConA. For the synthesis of surface functionalized particles, apparently optimization of the surface functionalization with respect to colloidal stability and minimal non-specific interactions appears to be more important than strategies to maximize the FG density, as the latter does not necessarily correlate directly with a higher number of surface-attached biomolecules.

## Conclusion and Outlook

In this work, we present a versatile set of cleavable probes (*N*-APPA and SPDP) for the quantification of derivatizable carboxy and amino surface groups on different types of nanoparticles. The straightforward catch-and release assays utilizing these probes enable the quantification of the cleaved-off reporters in the supernatant, and thus, circumvent interferences resulting from particle scattering as well as a sample-inherent absorption and emission of light. Thereby also often encountered fluorescence quenching can be bypassed. Validation of the results from the optical assays can be elegantly achieved by comparison of the optical assays with the determination of sulfur in the different supernatants using ICP-OES. Surface group quantification utilizing *N*-APPA and SPDP is not limited to PSP matrices, but can be extended to inorganic porous systems such as MSN. From the signal-to-noise ratios obtained for the photometric detection of the cleaved-off reporter unit 2-TP one can estimate the detection limits of our catch-and-release assay, which not only depends on the amount of sample, but also on the amount of FGs per particle. For particles with high FG densities, the amount of sample can most likely be reduced by at least a factor of ten compared to this work (here always 5 mg of NPs were applied in the FG quantification assays), while for NPs with a very low FG density, at maximum only a moderate reduction in sample amount might be feasible. However, if a fluorometrically detectable reporter unit is applied instead of colorimetric 2-TP, such as a BODIPY dye, the required sample amount can probably be further reduced due to the higher sensitivity of fluorescence detection compared to absorption measurements.

Our results underline the reliability and versatility of our probe design and the catch-and-release strategy. Our versatile concept can be used for surface group quantification on all types of transparent, scattering, absorbing, and/or fluorescent particles. The cleavable probes can be easily expanded to the determination of other bioanalytically relevant functional groups like maleimide or alkyne functionalities by the choice of the reactive group. Additionally, they can be adapted to other analytical techniques requiring different reporters, or to different types of linkers that can be quantitatively cleaved thermally, photochemically, or by pH, utilizing well-established chemistry, e.g. from drug delivery systems.

## Supplementary information

Supporting Information
