# Supplementary material for: Multimodal Cleavable Reporters for Quantifying Carboxy and Amino Groups on Organic and Inorganic Nanoparticles
Source: Sci Rep. 2019 Nov 26;9:17577. doi: 10.1038/s41598-019-53773-3 (PMC6879591; doi:10.1038/s41598-019-53773-3)
Supplement: Supplementary file 1 — Supporting Information [file 41598_2019_53773_MOESM1_ESM.pdf]

Supporting Information

Multimodal Cleavable Reporters for Quantifying Carboxy and Amino Groups on Organic and Inorganic Nanoparticles

Nithiya Nirmalananthan-Budau,[1,2] Bastian Rühle,[1] Daniel Geißler,[1] Marko Moser,[1,2] Christopher Kläber,[1] Andreas Schäfer,[2] Ute Resch-Genger[1,*]

[1)] Federal Institute for Materials Research and Testing (BAM), Richard-Willstätter-Str. 11, D-12489 Berlin, Germany
[2)] Institut für Chemie und Biochemie, Freie Universität Berlin, Takustrasse 3, 14195, Berlin, Germany
Corresponding Author* U.R.-G.: e-mail, ute.resch@bam.de; phone, ++49(0)30-8104-1134; fax, ++49(0)30-8104-71134.

<u>Table of Content:</u>

I Synthesis and Characterization of polystyrene nanoparticles

Figure S1: Reaction scheme for the synthesis of carboxy PSP **page 2**

Figure S2: Change of the particle-size with reaction time. **page 2**

Table S1: Characterization of carboxy PSP **page 3**

Figure S3: Derivatization scheme of carboxy PSP to amino PSP **page 3**

Table S2: Characterization of amino PSP **page 4**

II Synthesis and Characterization of mesoporous silica nanoparticles

Figure S4: Reaction scheme for the synthesis of MSN **page 5**

Table S3: Characterization of carboxy and amino MSN **page 5**

Figure S5: FTIR spectra of sample MSN-COOH-Hi **page 6**

Figure S6: Nitrogen sorption data of MSN samples **page 7**

Figure S7: TGA data of MSN samples **page 8**

III Conductometric titration of carboxylated and aminated PSP and MSN

Figure S8: Conductometric titration of carboxylated and aminated PSP **page 9**

Figure S9: Conductometric titration of carboxylated and aminated MSN **page 10**

IV Quantitative NMR of aminated and carboxylated MSN

Figure S10: qNMR spectrum of sample MSN-NH_2-Hi **page 11**

Figure S11: qNMR spectrum of sample MSN-NH_2-Lo **page 12**

Figure S12: qNMR spectrum of sample MSN-COOH-Hi **page 13**

Figure S13: qNMR spectrum of sample MSN-COOH-Lo **page 14**

V Amino and Carboxy Group quantification and validation of PSP

Figure S14: PSP-COOH-Me and PSP-COOH-Lo validation with ICP-OES **page 15**

Figure S15: FG quantification of PSP with multimodal cleavable reporters **page 15**

VI Amino and Carboxy Group quantification and validation of MSN

Figure S16: MSN-COOH-Lo validation with ICP-OES **page 15**

Figure S17: FG quantification of MSN with multimodal cleavable reporters **page 16**

VII Quantification of dye loaded carboxy PSP

Figure S18: Absorption and emission spectra of dyes in THF and the overlap of NHS-Fluorescein and Fluram **page 16**

VIII Biofunctionalization of carboxy PSP

Figure S19: Biomolecule-derivatizable amount of carboxylic functions **page 17**

I Synthesis and Characterization of polystyrene nanoparticles

Synthesis of carboxy polystyrene nanoparticles. The carboxylated PSP with different FG densities were synthesized via emulsion polymerization under argon atmosphere. For PSP core synthesis, a 400 µL aqueous solution of the radical starter PPS (0.148 mmol) was added to a mixture of 5200 µL of surfactant SDS (0.042 mmol) and 1300 µL of styrene monomer (11.36 mmol) in aqueous solution at 70°C. After 1 h of stirring at 350 rpm, 10 µL, 30 µL, 120 µL or 300 µL of co-monomer AA (0.146 mmol, 0.437 mmol, 1.75 mmol, 4.37 mmol) were added to the suspension and stirred for further 3 h at 70 °C. After cooling to room temperature (RT), the particle suspension was diluted 6-fold. To remove large chunks, the suspension was centrifugated two times at 15000 g for 2 min and the supernatants were collected. For all further experiment the combined supernatant was used after three further centrifugation and washing steps at 16000 g for 1 h for removing the SDS and KPS containing supernatant.

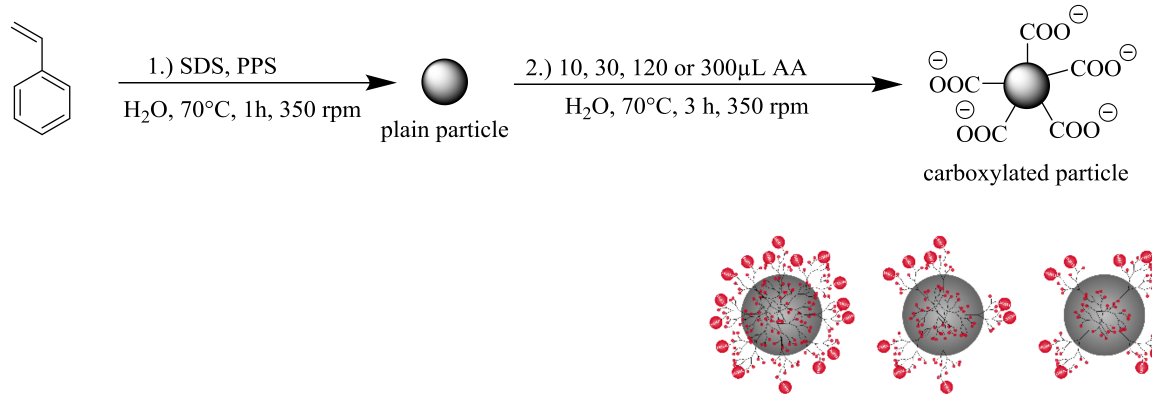

Figure S1: Reaction scheme for the synthesis of carboxy polystyrene nanoparticles with different densities of FGs.

Synthesis screening of carboxy polystyrene nanoparticles. At 70°C the core particles are generated within one hour. By adding different amounts of co-monomer acrylic acid (AA) the surface of the plain core will be grafted with different densities of carboxy functional groups (FGs). The core-shell particles synthesis was quenched by dilution with 35 mL of water after 240 min since the hydrodynamic diameter of the particle did not change significantly any more after 180 min.

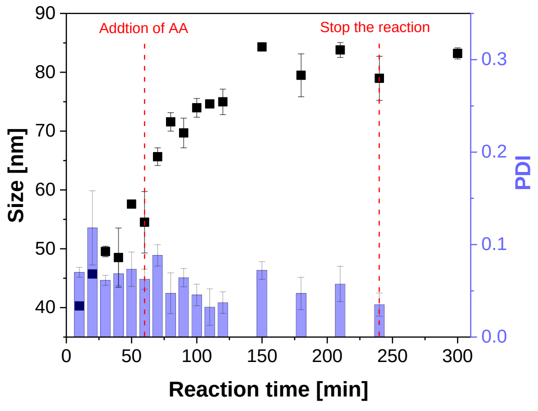

Figure S2: Change of particle size with reaction time.

Characterization of carboxy PSP. The NP with different amounts of co-monomer AA have a hydrodynamic diameter of around 110 nm. While addition of high amounts of AA leads to a polydisperse particle suspension with core-shell particles and smaller acrylic acid particles (see Table S1 last column), a low volume of co-monomer leads to a colloidally unstable core-shell system in which the particles tend to agglomerate and show a higher hydrodynamic diameter and PDI. As expected, the zeta potential decreased with increasing FG density.

Table S1: Characterization of carboxy PSP.

	**PSP-COOH**			
	**10 µL AA (PSP-COOH-Lo)**	**30 µL AA (PSP-COOH-Me)**	**120 µL AA (PSP-COOH-Hi)**	**300 µL AA**
**Hydrodynamic diameter [nm]**	148 ± 27	121 ± 1	113 ± 3	polydisperse
**PDI**	0.280 ± 0.033	0.090 ± 0.027	0.142 ± 0.009	-
**Zeta potential**	-25 mV	-30 mV	-48 mV	-
**SEM**	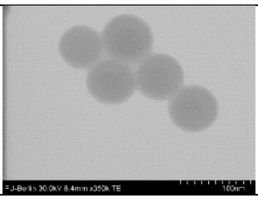	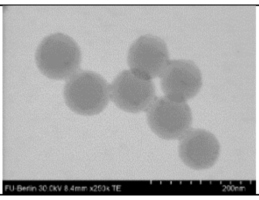	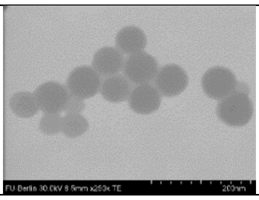	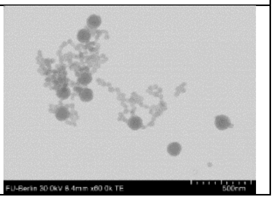

Synthesis of aminated polystyrene nanoparticles. The aminated PSP with different FG densities were synthesized by derivatization of the carboxy PSP with the highest FG density. 120 µL of EDC (150 mM) and 60 µL of s-NHS (300 mM) in MES buffer (0.05 M; pH 5) were added to 100 µL of carboxylated PSP (5 wt %) after washing the particle three times with MES buffer. The mixture was shaken at 600 rpm for 1 h at RT, followed by one washing step using 480 µL of phosphate buffer (0.01 M, pH 8). Subsequently, 580 µL of the activated carboxylated particle suspension (0.86 wt-%) were added to a solution of 200, 1000, 2500 nmol *N*-Boc-ethylendiamine dissolved in 20 µL of DMSO. The reaction mixture was shaken at 600 rpm for 16 h at RT, followed by centrifugation at 16,000 g for 40 min. The supernatant was removed, and the PSP were deprotected two times with 400 µL of HCl (0.085 M) and washed two times with phosphate buffer (0.01 M, pH 8).

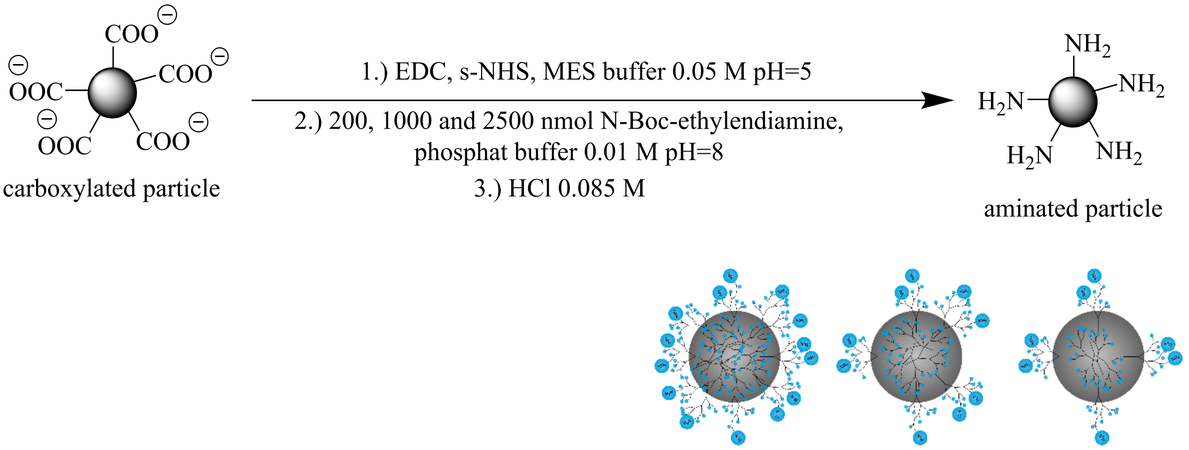

Figure S3: Derivatization scheme of carboxy PSP to amino PSP.

Characterization of amino PSP.

Table S2: Characterization of amino PSP.

	PSP-NH_2		
	**200 nmol EDA (Low)**	**1000 nmol EDA (Medium)**	**2500 nmol EDA (High)**
**Hydrodynamic diameter [nm]**	108 ± 3	124 ± 4	135 ± 5
**PDI**	0.115 ± 0.009	0.148 ± 0.011	0.130 ± 0.012
**Zeta potential**	-32 mV	-31 mV	-25 mV

II Synthesis and Characterization of mesoporous silica nanoparticles (MSN)

Synthesis of aminated MSN. For the synthesis of aminated MSN with different FG densities, a mixture of 100 mL of Millipore water, 200 mg of hexadecyltrimethylammonium bromide (CTAB; 0.549 mmol) and 1200 µL of NaOH (1.00 M) was prepared and stirred for 20 min @ 80°C. Then, a mixture of 1000 µL of tetraethoxysilane (TEOS; 4.48 mmol) and an appropriate amount of 3-aminopropyl triethoxysilane (APTES; 0.1 mol% or 10 mol%; 1.06 µL or 106 µL) was added dropwise to this mixture under vigorous stirring. The resulting mixture was vigorously stirred for another 2 h at 80°C and afterwards centrifuged for 12 min at 13,640 g. The supernatant was discarded, and the nanoparticles were washed twice with EtOH (2 x 90 mL). To extract the surfactant from the pores, the nanoparticles were resuspended in 90 mL of ethanolic NH_4NO_3 solution (20 mg/mL), refluxed for 2 h, centrifuged again for 12 min at 13,640 g, washed once with 90 mL of EtOH, resuspended in 90 mL of EtOH:HCl (conc) (90:10 v/v) and refluxed again for 2 h. Finally, the suspension was centrifuged for 12 min at 13,640 g, washed twice with EtOH (2 x 90 mL) and stored in 30 mL of EtOH.

Synthesis of carboxylated MSN. For the synthesis of carboxylated MSN with different FG densities, a mixture of 100 mL of Millipore water, 200 mg of hexadecyltrimethylammonium bromide (CTAB; 0.549 mmol) and 1200 µL of NaOH (1.00 M) was prepared and stirred for 20 min @ 80 °C. Then, a mixture of 1000 µL of tetraethoxysilane (TEOS; 4.48 mmol) and an appropriate amount of 2-cyanoethyl triethoxysilane (CETES; 0.1 mol% or 10 mol%; 1.01 µL or 101 µL) was added dropwise to this mixture under vigorous stirring. The resulting mixture was vigorously stirred for another 2 h at 80°C and afterwards centrifuged for 12 min at 13,640 g. The supernatant was discarded, and the nanoparticles were washed twice with EtOH (2 x 90 mL). To extract the surfactant from the pores, the nanoparticles were resuspended in 90 mL of ethanolic NH_4NO_3 solution (20 mg/mL), refluxed for 2 h, centrifuged again for 12 min at 13,640 g, and washed once with 90 mL of EtOH. To further extract the template and to hydrolyze the nitrile groups to carboxylic acid groups, the particles were resuspended in 40 mL of H_2O:HCl (conc) (10:30 v/v) and refluxed for 5 h. Successful hydrolysis of the nitrile groups to carboxy groups was confirmed from ATR-IR spectroscopy (Figure S5; after hydrolysis, new bands appear at ν = 1718 cm^{-1} and ν = 1412 cm^{-1} that can be assigned to C=O stretching and O-H bending vibrations, respectively) and NMR spectroscopy (Figure S10 and S11; the signal at δ = 2.44 ppm from the cyanoethylsilane (peak labeled with a # sign) is negligible after hydrolysis, and a new peak at δ = 2.09 ppm (peak labeled c) that can be assigned to the methylene group of the carboxy-silane is clearly visible). Finally, the suspension was centrifuged for 12 min at 13640 g, washed twice with EtOH (2 x 90 mL) and stored in 30 mL of EtOH.

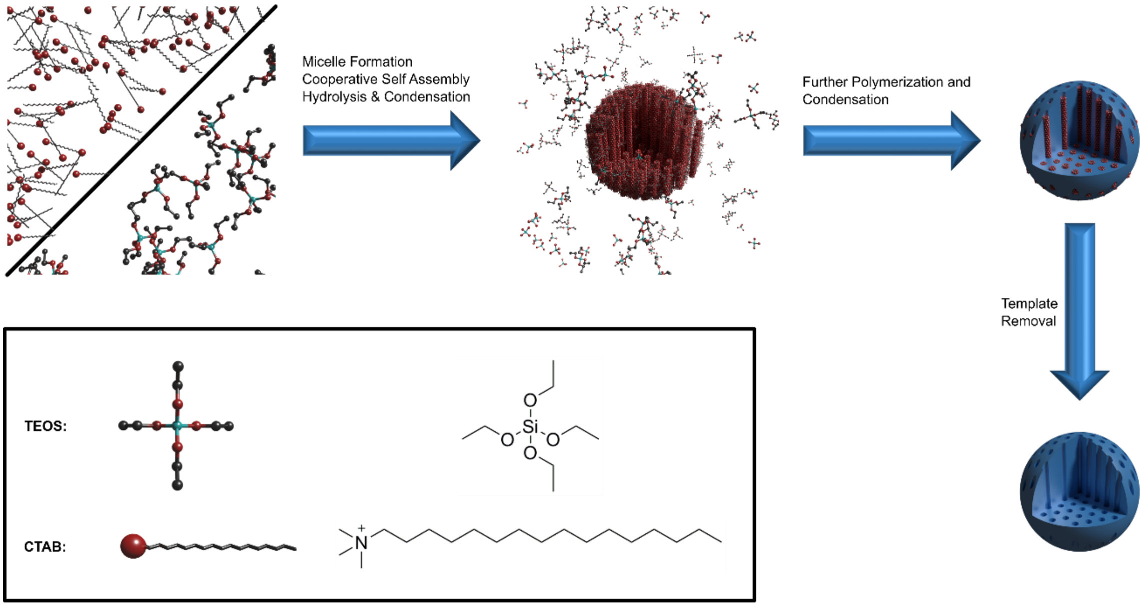

Figure S4: Reaction scheme for the synthesis of mesoporous silica nanoparticles.

Characterization of aminated and carboxylated MSN.

Table S3: Characterization of aminated and carboxylated MSN.

	MSN-COOH		$MSN\text{-}NH_2$	
	**0.1 mol% (low)**	**10 mol% (high)**	**0.1 mol% (low)**	**10 mol% (high)**
**Hydrodynamic diameter [nm]**	252.0 ± 8.9	222.3 ± 1.7	320.6 ± 2.7	216.5 ± 1.8
**PDI**	0.257 ± 0.010	0.237 ± 0.013	0.228 ± 0.014	0.226 ± 0.006
**Zeta potential [mV]**	-25.1 ± 3.0	-15.4 ± 1.63	+34.3 ± 1.71	+41.3 ± 0.86
**TGA weight loss at 700°C [wt %]**	5.4	9.1	10.9	14.4
**S_{BET} [m²/g]**	738	729	1033	976
**V_{tot} [cm³/g]**	1.12	1.23	1.58	1.88
**d_{BJH} [nm]**	2.4	2.3	2.5	2.5
**d_{NLDFT} [nm]**	3.7	3.4	4.4	4.3

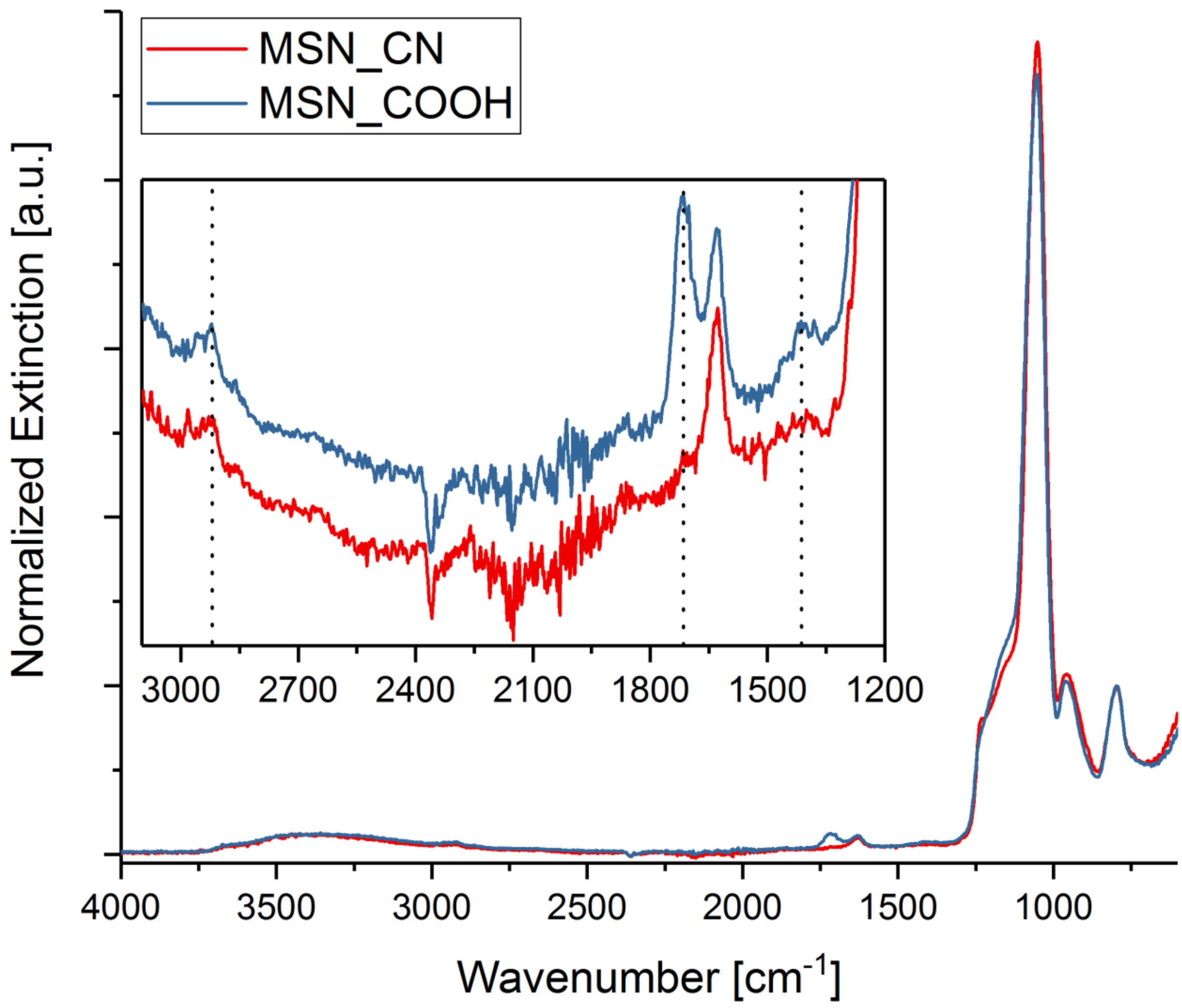

Figure S5: FTIR spectra of sample MSN-COOH-Hi before (red) and after (blue) hydrolysis of the nitrile groups of CETES to carboxy groups. Spectra are normalized to the Si-O-Si vibration at ν = 796 cm^{-1} and slightly vertically offset for clarity. Dotted lines in the inset indicate the C-H stretching vibrations at around 2923 cm^{-1}, C=O stretching vibrations at ν = 1718 cm^{-1}, and O-H bending vibrations at ν = 1412 cm^{-1}, respectively.

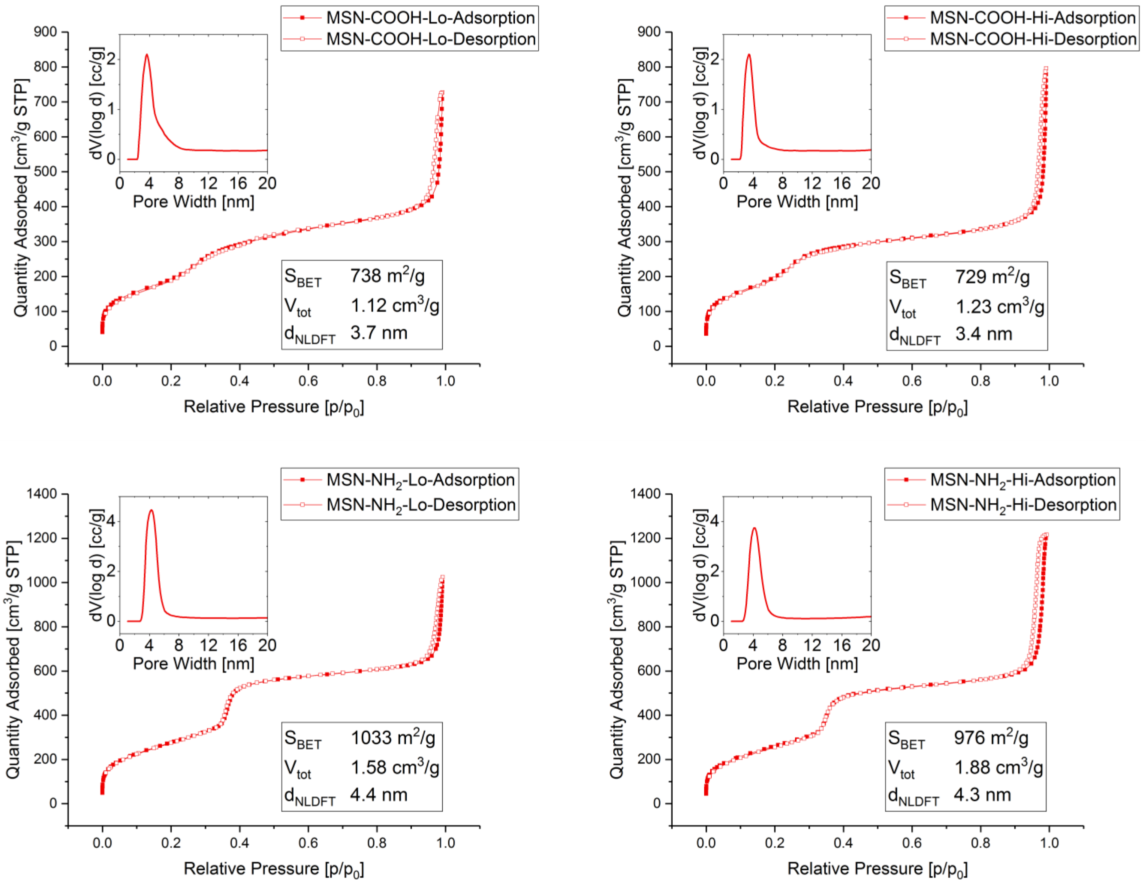

Figure S6: Nitrogen sorption data of samples MSN-COOH-Lo (top left), MSN-COOH-Hi (top right), MSN-NH_2-Lo (bottom left), MSN-NH_2-Hi (bottom right). Insets show the NLDFT pore size distribution. The BET surface area (S_{BET}), the total pore volume (V_{tot}), and the NLDFT pore diameter at the maximum of the pore size distribution (d_{NLDFT}) are also indicated.

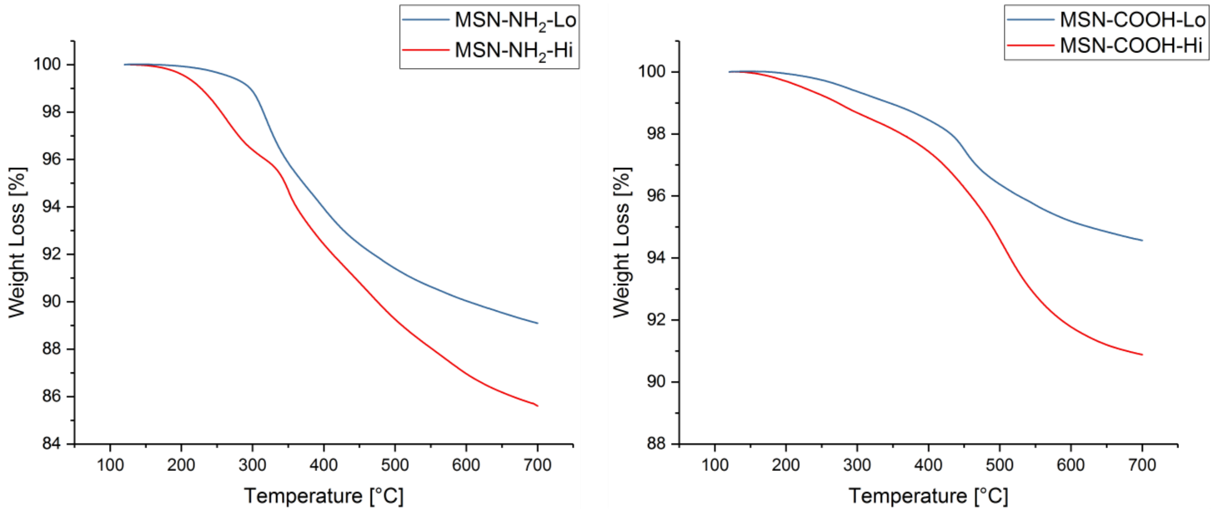

Figure S7: Thermogravimetric analysis of samples MSN-NH_2-Lo and MSN-NH_2-Hi (left) and MSN-COOH-Lo and MSN-COOH-Hi (right).

III Conductometric titration of carboxylated and aminated PSP and MSN

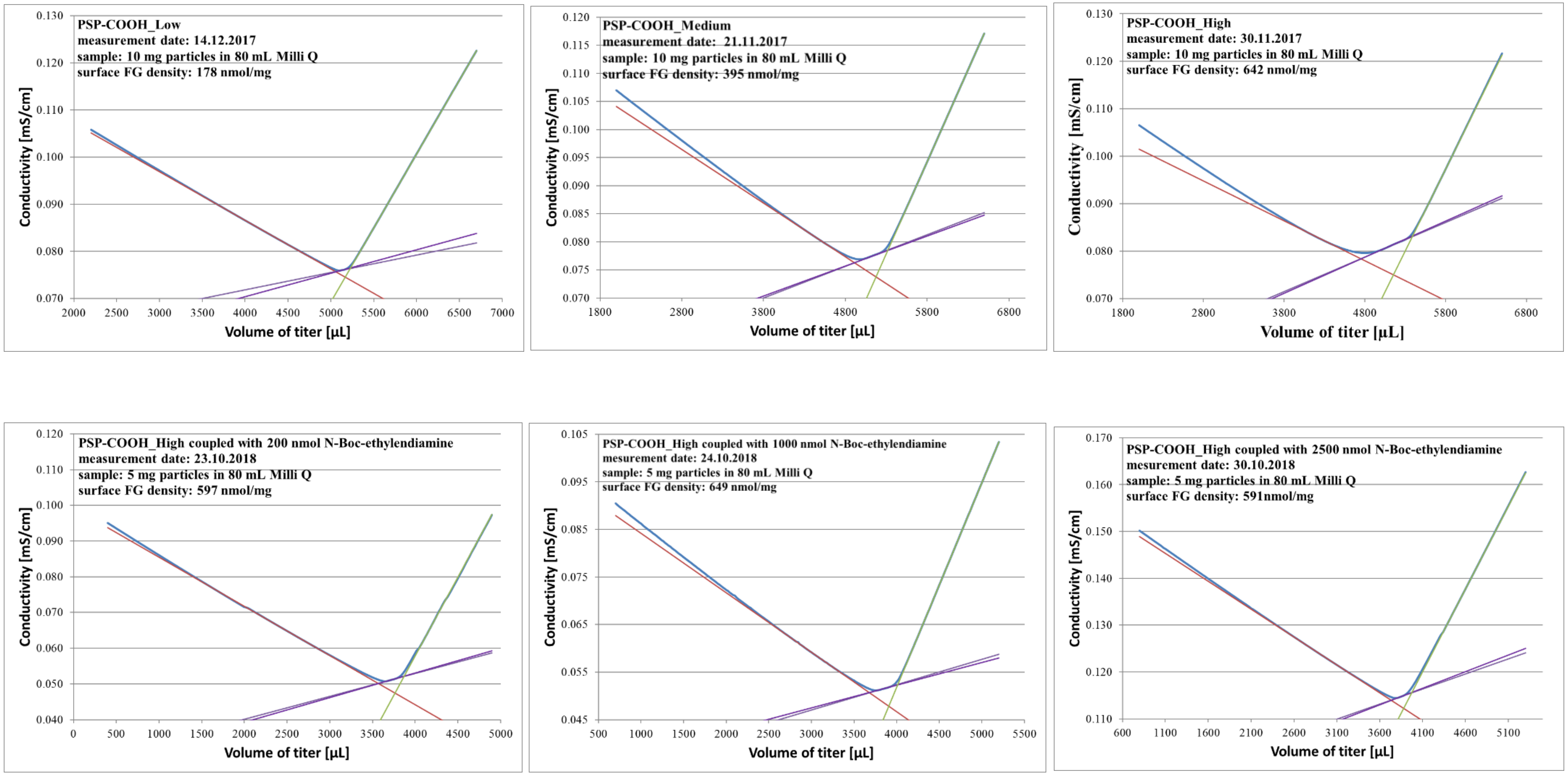

Figure S8: Conductometric titration of carboxylated (top) and aminated (bottom) PSP; 10 mg of PSP-COOH-Lo (top, left), PSP-COOH-Me (top, middle), PSP-COOH-Hi (top, right) and 5 mg of PSP-NH_2-Lo (bottom, left), PSP-NH_2-Me (bottom, middle), PSP-NH_2-Hi (bottom, right) were titrated for the quantification of the total amount of FGs.

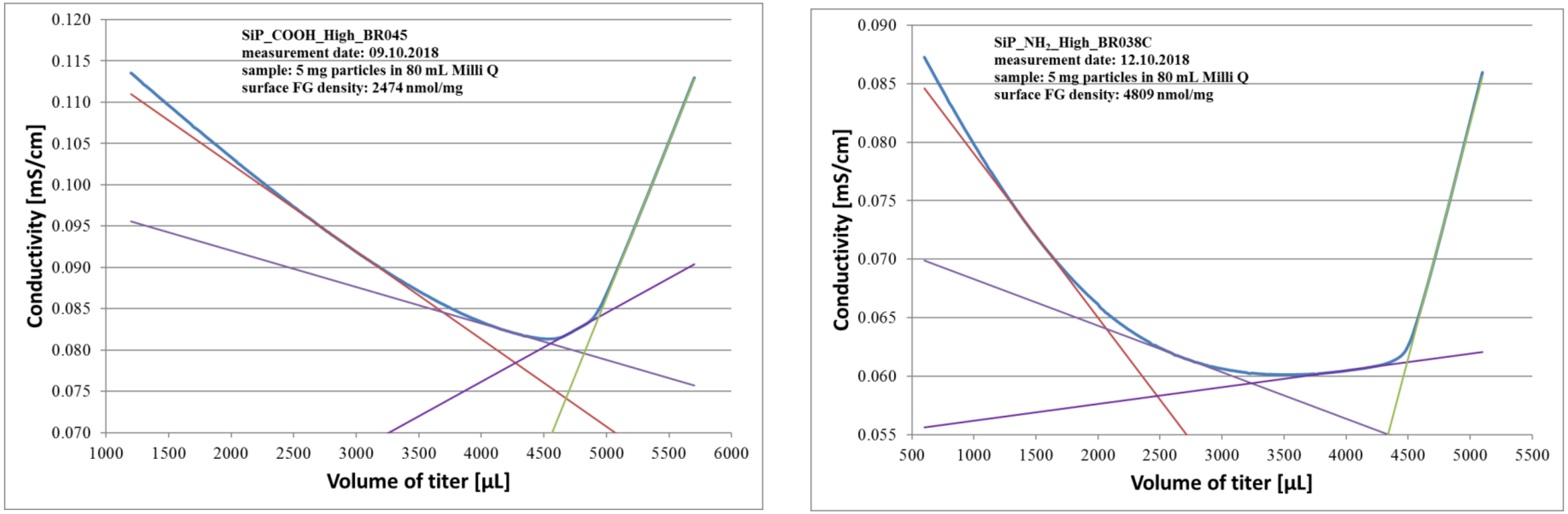

Figure S9: Conductometric titration of carboxylated (left) and aminated (right) MSN.

IV Quantitative NMR of aminated and carboxylated MSN

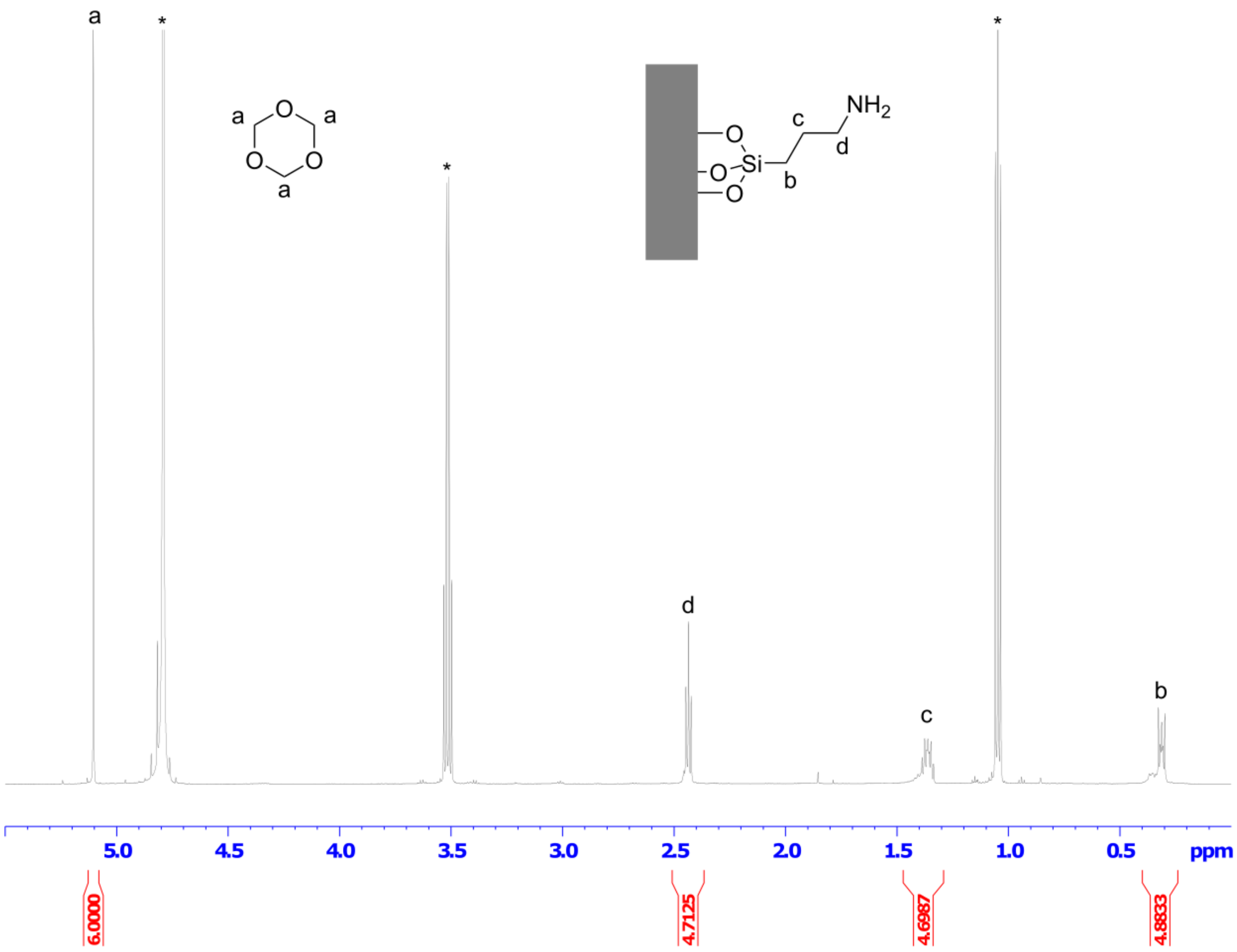

Figure S10: qNMR spectrum of sample MSN-NH_2-Hi. Signals indicated with an asterisk are from the solvent and EtOH.

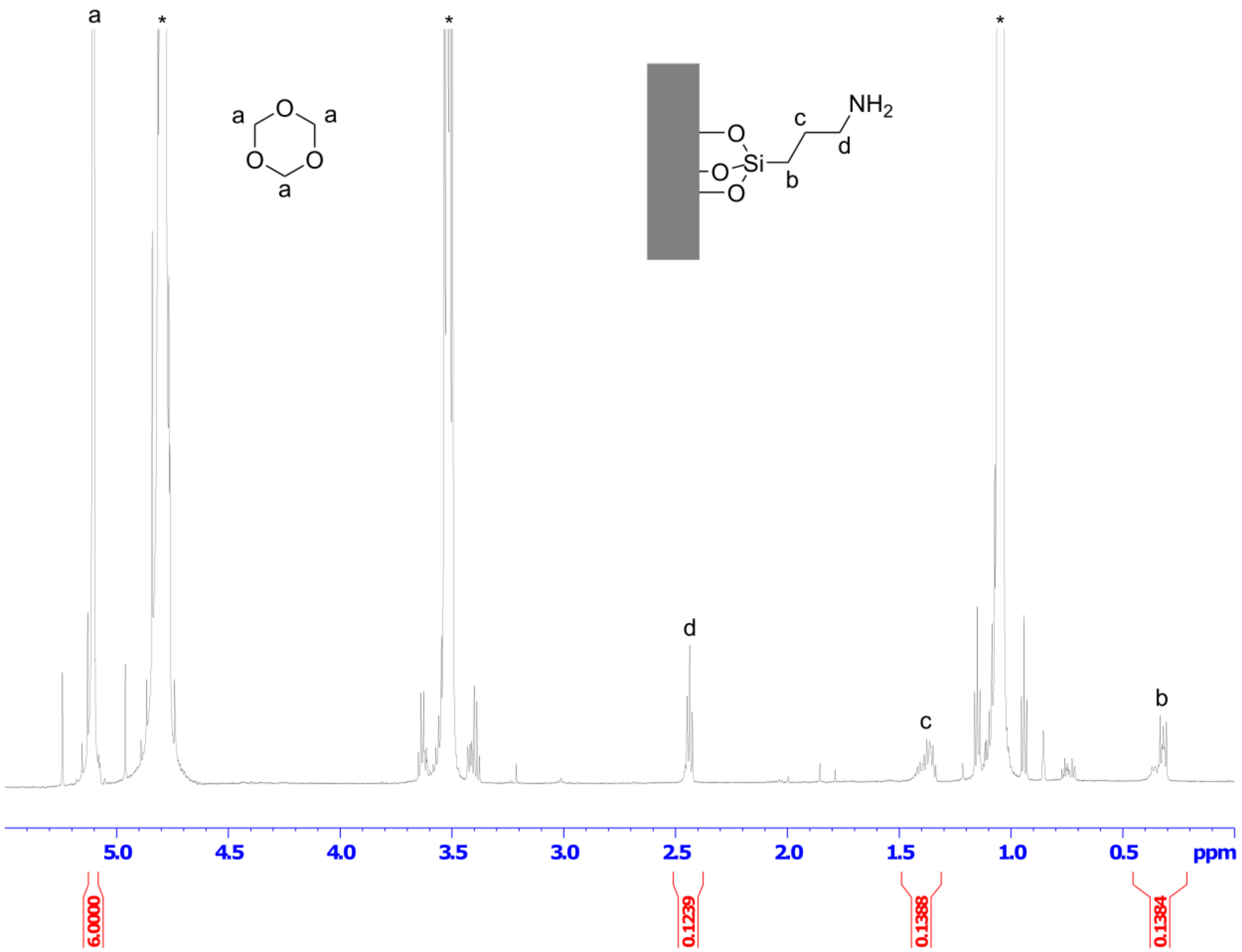

Figure S11: qNMR spectrum of sample MSN-NH_2-Lo. Signals indicated with an asterisk are from the solvent and EtOH.

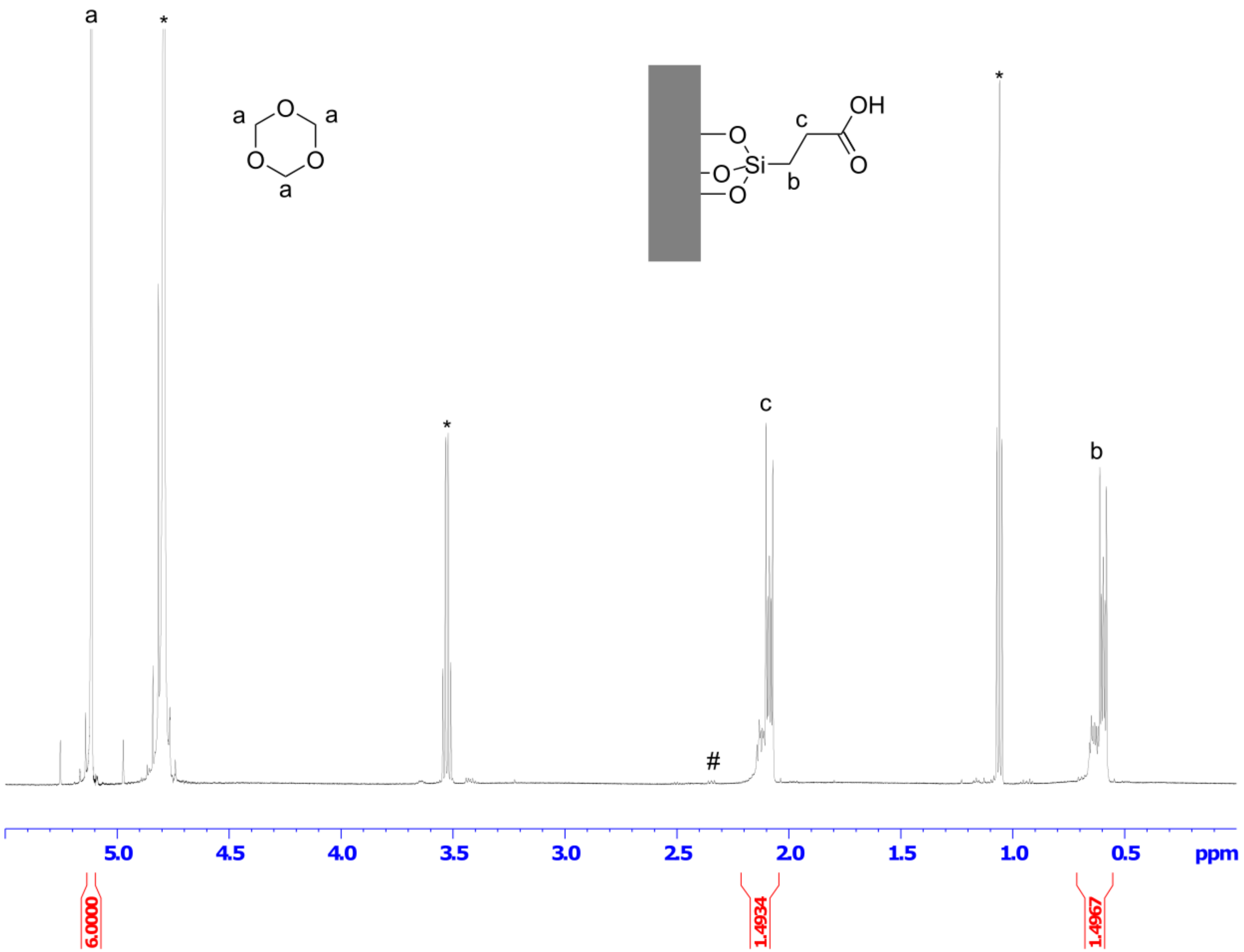

Figure S12: qNMR spectrum of sample MSN-COOH-Hi. Signals indicated with an asterisk are from the solvent and EtOH. The signal indicated with a pound sign can be assigned to the unhydrolyzed precursor (cyanoethylsilane).

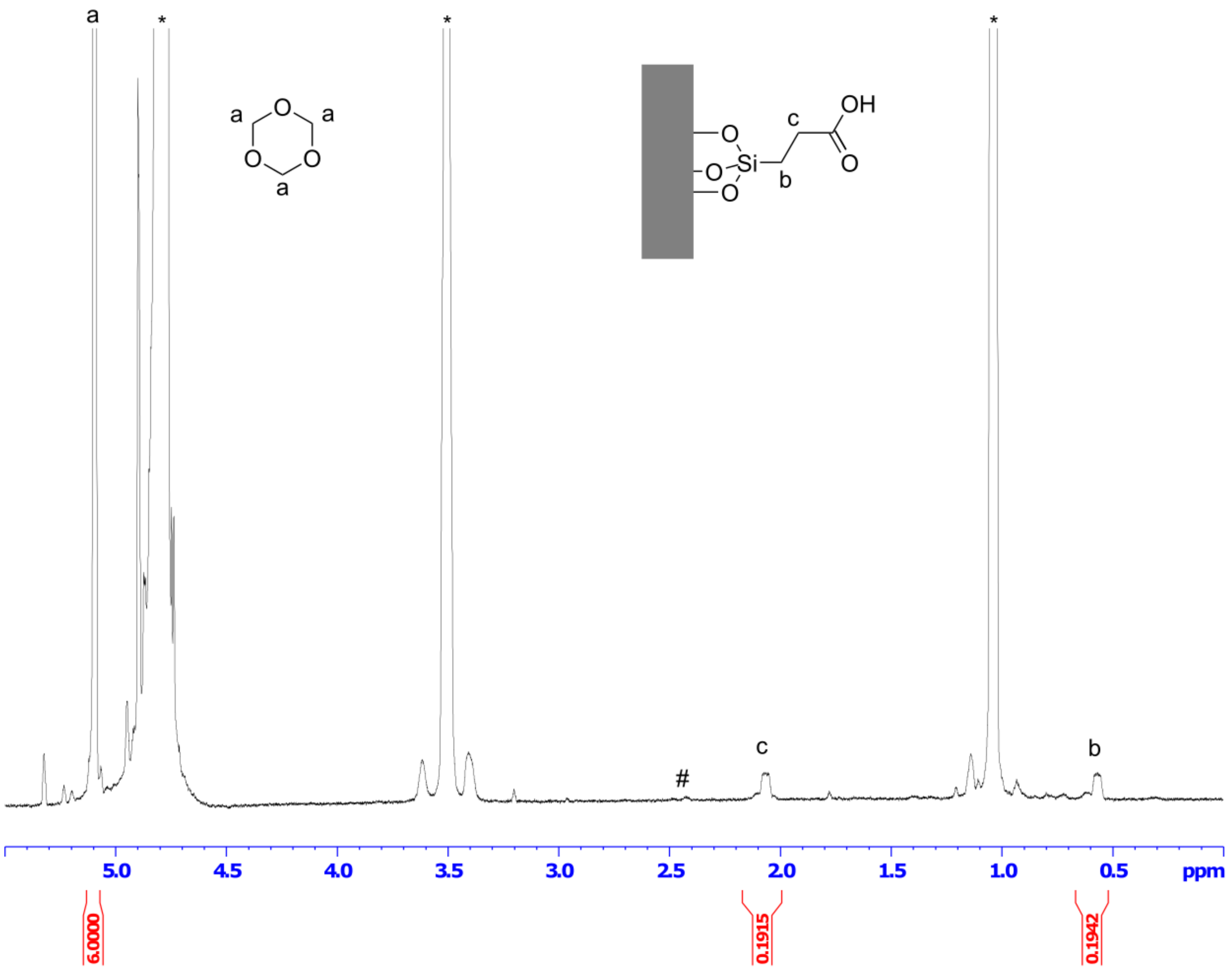

Figure S13: qNMR spectrum of sample MSN-COOH-Lo. Signals indicated with an asterisk are from the solvent and EtOH. The signal indicated with a pound sign can be assigned to the unhydrolyzed precursor (cyanoethylsilane).

V Amino and Carboxy Group quantification and validation of PSP

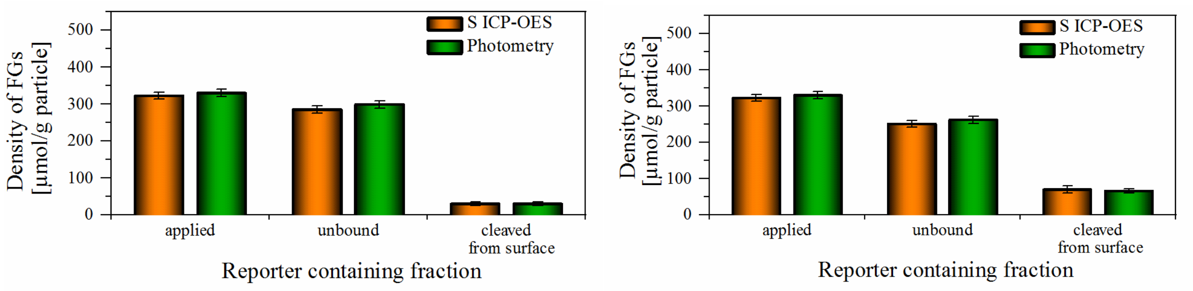

Figure S14: Validation of surface group analysis with *N*-APPA using ICP-OES for low and medium carboxy PSP.

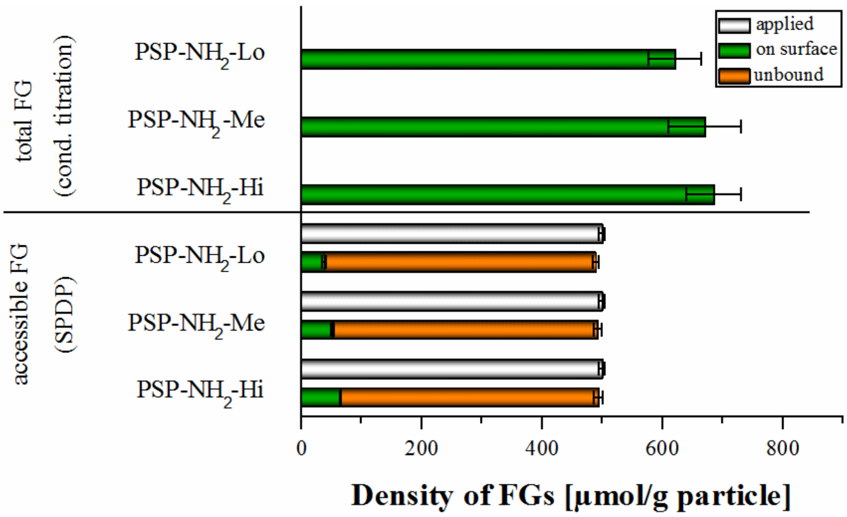

Figure S15: FG quantification with conductometric titration and multimodal cleavable reporter SPDP on self-synthesized amino PSP of different surface functional group densities.

VI Amino and Carboxy Group quantification and validation of MSN

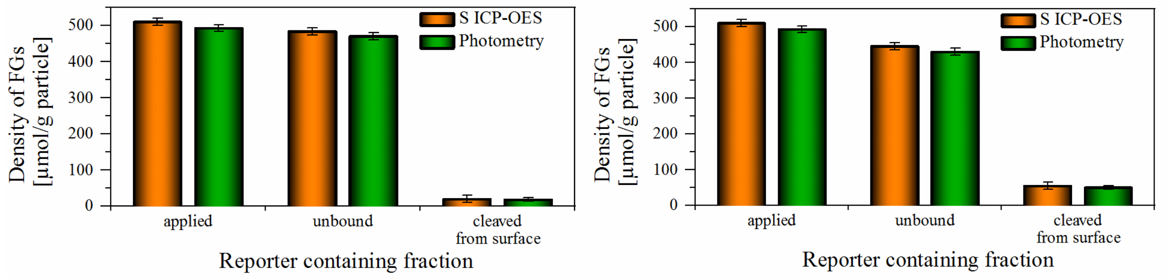

Figure S16: Validation of surface group analysis with *N*-APPA using ICP-OES for low and high carboxy MSN.

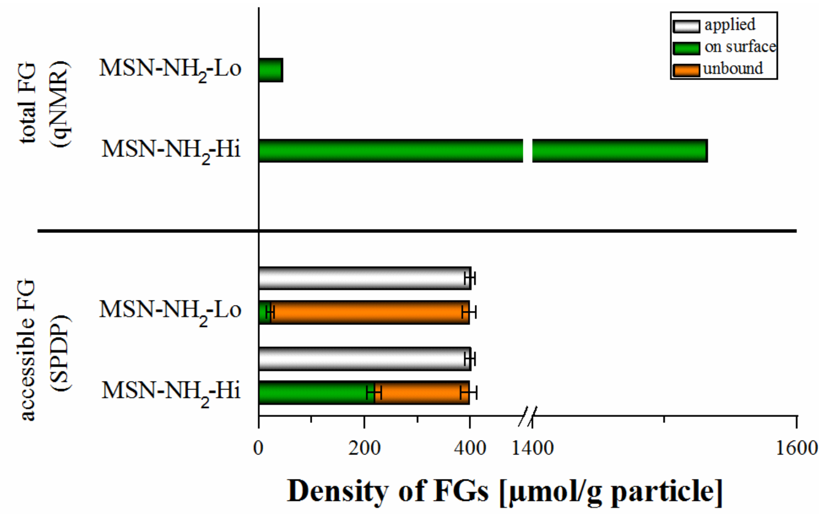

Figure S17: FG quantification with multimodal cleavable reporter SPDP on self-synthesized amino MSN of two different surface functional group densities.

VII Quantification of dye loaded carboxy PSP

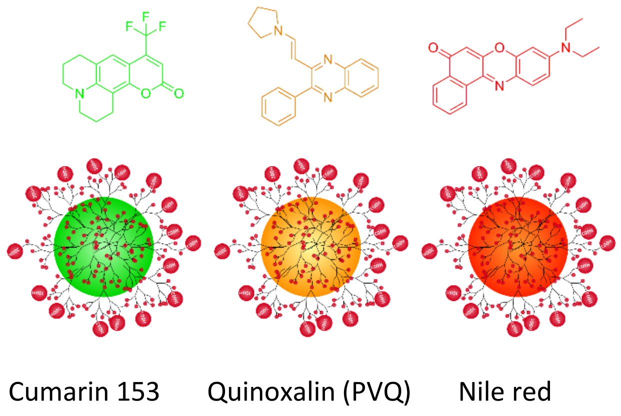

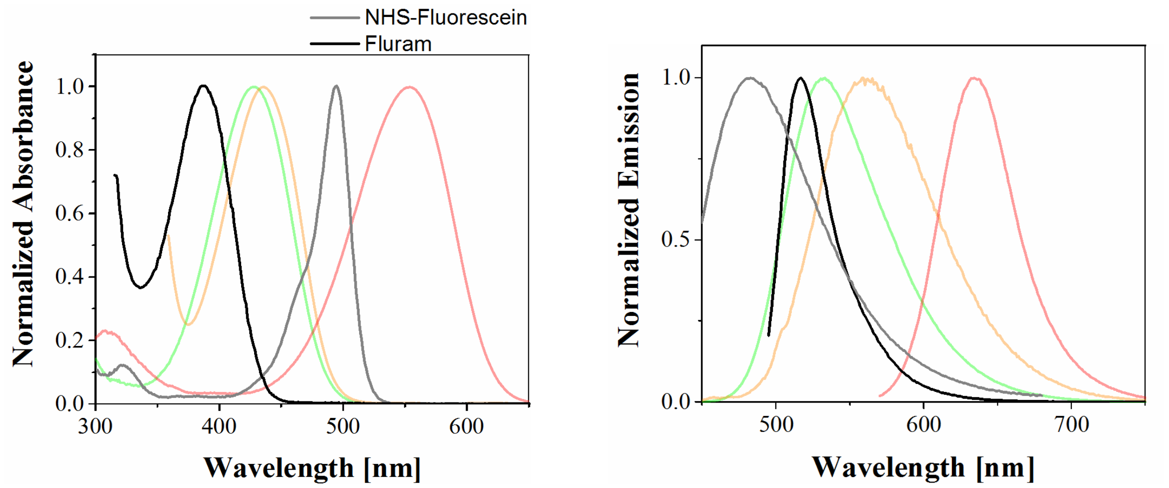

Figure S18: Absorption (left) and emission spectra (right) of Cumarin 153, Nile red and PVQ in THF and the overlap of NHS-Fluorescein and Fluram labels.

VIII Biofunctionalization of carboxy PSP

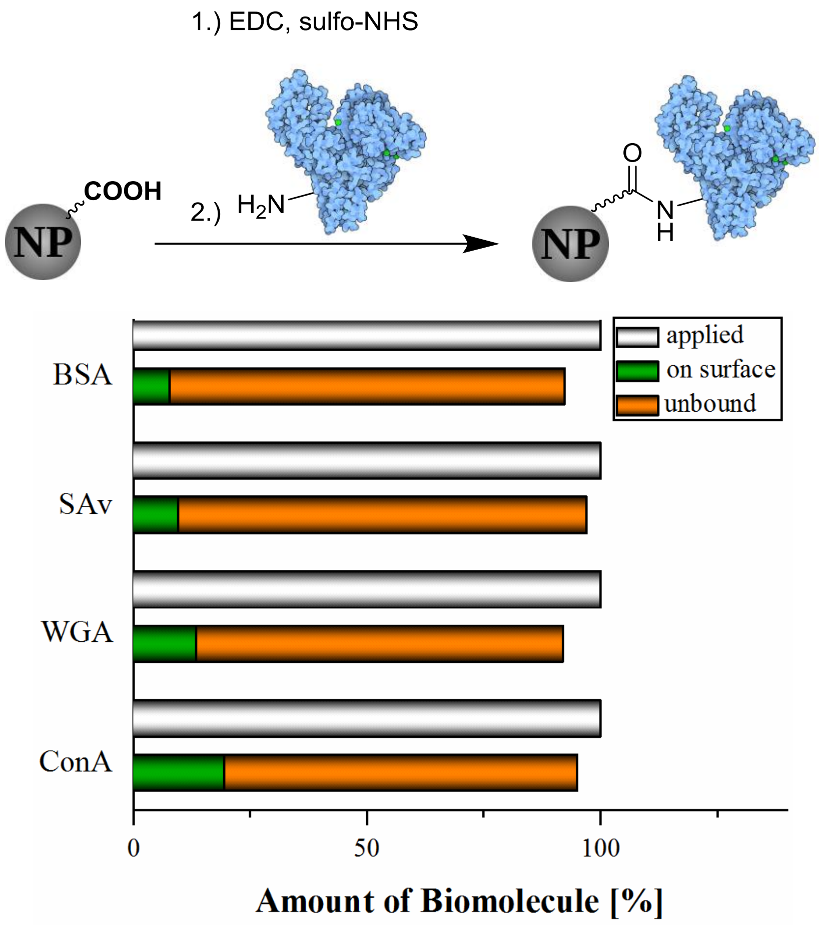

Figure S19. Biomolecule-derivatizable amount of carboxy functions obtained for four differently sized proteins.
